# Letter from E. Andrews, M. D.

**Published:** 1862-11

**Authors:** E. Andrews

**Affiliations:** Army in the Field, South of Tallahatchie River, Mississippi


### Army Correspondence.

ARMY IN THE FIELD, SOUTH OF TALLAHATCHIE RIVER, MISSISSIPPI, *Dec.* 5, 1862.

MESSRS. EDITORS:—Although there are no great combats to record at present, nevertheless, it may be of some interest to your readers to hear from this part of the army.

During the spring and summer, the most common diseases among us were diarrhœa and dysenteries, often running to the chronic form. As summer advanced, bowel diseases became a little less frequent, and periodical fevers took their place. In September, intermittents and remittents were considerably prevalent. A number of fatal cases of congestive fever occurred in the infantry, outside of Memphis, but scarcely any in the garrison proper. A few cases of remittent fever ran rapidly to a fatal termination; but scarcely any true typhoid fever proper, as seen in Chicago, occurred. September proved the sickliest month in the year, yet the number under treatment was only nine per cent of the whole force; which is one per cent less than Lord Wellington states as the minimum amount of sickness obtainable under the best circumstances. It was observable also, that the artillery suffered much less than the infantry; of about 175 cases of acute dysentery, occurring in the artillery during seven months, not one proved fatal, and sickness of other kinds was milder, and less frequent by far among them, than among infantry. I attribute the difference to the fact, that the infantry soldier on the march, is put to excessive exertion, both in carrying his knapsack, arms, and ammunition, and also by the very act of walking under a boiling southern sun. This aggravates all bowel complaints. When not on the march, he relapses into a state of too great inaction, having often for weeks very little vigorous exercise to perform; thus, alternately between the extremes of exhausting labor and enervating quietness, he suffers by the sudden changes to which he is exposed. The artillery soldier, on the contrary, has a pretty uniform life of moderate steady labor: the constant care of his horses, harness, side arms, etc. etc., secures steady exercise in camp, and when put upon he march, he rides either on horse or caisson, so that the transfer to the field makes very little increase of his hardships, or change in his mode of life. This state of things, very much favors a uniform good health.

In the cavalry I find that surgical accidents are very common, and especially for hernia. The act of riding brings the abdominal muscles into constant use in preserving the position of the body in the saddle, hence, the abdominal viscera being constantly pressed upon, at length bulge through the inguinal rings. Some also are kicked by horses; bruised on the pommel of the saddle, or fracture limbs by being thrown.

Our advance thus far into Mississippi has resulted in very little fighting. The front skirmished with slight loss; and there is little of surgical interest to relate. At present the diseases are few and sparse: consisting mainly of acute bronchitis, mild pneumonias, and slight rheumatics. We live on the country, finding plenty of poultry, cattle, sweet potatoes, corn, and forage, so that the diet both of men and horses is superb; and, of course, scurvey is out of the question, and other diseases mild. The climate is mild, generally requiring no overcoats in the day time, but giving us frequently a sharp frost at night. Our sick were left behind when we left Memphis, so that surgeon's duties are now light. The flora of this region presents some striking changes as we advance. The mistletoe is abundant, of course, and numerous other evergreens present themselves as we advance. Among them is the cane, which does not here get its full growth, but appears larger, as we advance. There are several woody vines which appear to retain their foliage all winter, and a number of trees of the latter one or two are, I believe, species of the holly; the trunk and leaf closely resemble the beech, but the foliage is evergreen, and present at the present time a vividness of green, not one whit less than that of the fullest glory of midsummer.

At present, this part of the army has halted south of the Tallahatchie, while the other is pursuing the flying enemy beyond Grenada. Apparently, we must do some weary marching before we can bring them again to battle. Yours truly.

E. ANDREWS.

